# Detection of Volatile Organic Compounds by Self-assembled Monolayer Coated Sensor Array with Concentration-independent Fingerprints

**DOI:** 10.1038/srep23970

**Published:** 2016-04-05

**Authors:** Ye Chang, Ning Tang, Hemi Qu, Jing Liu, Daihua Zhang, Hao Zhang, Wei Pang, Xuexin Duan

**Affiliations:** 1 State Key Laboratory of Precision Measuring Technology & Instruments, College of Precision Instrument and Opto-electronics Engineering, Tianjin University, Tianjin 300072, China

## Abstract

In this paper, we have modeled and analyzed affinities and kinetics of volatile organic compounds (VOCs) adsorption (and desorption) on various surface chemical groups using multiple self-assembled monolayers (SAMs) functionalized film bulk acoustic resonator (FBAR) array. The high-frequency and micro-scale resonator provides improved sensitivity in the detections of VOCs at trace levels. With the study of affinities and kinetics, three concentration-independent intrinsic parameters (monolayer adsorption capacity, adsorption energy constant and desorption rate) of gas-surface interactions are obtained to contribute to a multi-parameter fingerprint library of VOC analytes. Effects of functional group’s properties on gas-surface interactions are also discussed. The proposed sensor array with concentration-independent fingerprint library shows potential as a portable electronic nose (e-nose) system for VOCs discrimination and gas-sensitive materials selections.

Volatile organic compounds (VOCs) are carbon based chemicals that easily emit from industry productions or indoor environments (*e.g.* furnishings, paints and building materials) which are due to their rather low boiling points. VOCs are believed to have short-term and long-term adverse effects on environment and human health, including the potential cause of cancer^1^,^2^,^3^. Moreover, some exhaled VOCs are found as effective biomarkers that could be used for the detection of some diseases, including lung cancer. Thus, there is a large demand on the sensitive and selective detection of VOCs in the gas phase for environmental monitoring, process control, and medical diagnostics purposes^4^,^5^,^6^,^7^,^8^. Accordingly, these applications prefer to have portable sensors which can on-site predict the contents of VOC mixtures and concentration information of each composition. However, most commercial potable VOC sensors in the current market (*e.g.*, photoionization sensors and metal oxide sensors) are dedicated to concentration detections, which leads to a problem of discriminating individual analyte in a vast VOC spectrum. An alternatively useful method is the electronic nose (e-nose) system that consists of an array of chemical gas sensors^9^,^10^,^11^. Compared with individual sensor detection, the high-integrated sensor array satisfies the needs of the discrimination of VOCs and determination their concentrations.

In general, each component of the e-nose array is chemically modified with different gas-sensitive materials, providing a response spectrum, *i.e.*, the “fingerprint”^12^,^13^. Conventional fingerprints are based on direct sensor response (single parameter) to a known concentration of the analyte^14^. Different VOCs may appear similar fingerprint patterns thus, the discrimination capability of the single parameter fingerprints is limited. Multiple parameters fingerprints have been recently reported using silicon nanowire field effect transistors functionalized with different self-assemble monolayers (SAMs)^15^,^16^. Multiple sensor output (voltage threshold, on-current, hole mobility and subthreshold swing) for VOCs detections were recorded to generate multiple fingerprint patterns which largely enhance the discriminative power of such e-nose system^17^,^18^,^19^,^20^. The responses from the nanowire sensors are generally caused by the polarity of the gas molecules, which do not show direct correlation with the gas concentrations. Thus, they cannot be fitted directly by the known gas-solid surface adsorption isotherms. The fingerprint patterns are built directly from the sensor output which is dependent on the VOC concentrations. Thus, the applied condition of the device is limited (*e.g.,* an analyte with unknown concentration is difficult to be distinguished with this approach). Hence, a concentration-independent discrimination method is urgently needed. The interfacial chemi(physi)sorption parameters (*e.g.* affinities and kinetics constant) of VOCs interaction with functional sensor surface are specific and concentration independent^21^. These constants reflect the intrinsic information of VOCs at different gas-solid interface and in principle, can be used as multi-parameter fingerprints with high discriminative power (Fig. 1).

One class of sensors with the potential to be a candidate for such multi-parameter determinations is the acoustic wave resonators^22^,^23^. Based on gravimetric detections, the sensor output is directly linear to the amount of adsorbed gas molecule and real-time sorption process can be recorded as well. Thus, intrinsic interaction information, *e.g.*, affinities and kinetics of VOCs adsorption onto various functionalized surfaces can be analyzed with different isotherms and kinetic models. To date, a number of attempts for gas sensing with acoustic wave resonators, particularly surface acoustic wave (SAW) device^24^,^25^,^26^,^27^, quartz crystal microbalance (QCM)^28^,^29^,^30^,^31^,^32^,^33^ and cantilever^34^,^35^ have been made with remarkable results. However, the operation frequencies of those devices are limited (below GHz), and could not meet the demand of high sensitivity for vapor detections at trace levels which is normally required for the analysis of the adsorption affinity and kinetics parameters. With operation frequency extended to a few GHz, sensor based on film bulk acoustic resonator (FBAR) shows much higher sensitivity in the field of chemical and biological detections^36^ and has yielded several progresses in gas sensing applications^37^,^38^,^39^,^40^,^41^,^42^. The limit of detection (LOD) of FBAR is in the sub-picogram level which is much less than the mass loading of a single layer of gas molecules. Moreover, it is especially noteworthy benefiting from their micrometer-scale size and CMOS-compatible fabrications, FBARs can be easily integrated into sensor array as portable e-nose system^43^,^44^.

In this work, we fabricated high working frequency FBAR sensors (4.44 GHz), and coated individually with different SAMs. E-nose type gas sensors were fabricated by integrating different chemical functionalized FBAR into arrays. Adsorption affinities and kinetics parameters (monolayer adsorption capacity, adsorption energy constant and desorption rate constant) of five VOCs (methanol, ethanol, n-propanol (NPA), i-propanol (IPA) and acetone) interactions with these functional sensors are modeled to provide a multiple concentration-independent fingerprint library, offering a more direct and accurate method for VOCs discriminations. A special focus is on understanding the characteristics of different SAMs in terms of their hydrophilicities, length of the SAMs, functional group densities as effective factors to discriminate VOCs adsorption which is targeted for gas-sensitive materials selections.

## Results

### Film bulk acoustic resonator

For the acoustic bulk wave resonators used as mass sensors, Sauerbrey first developed an equation to describe the relationship between the change in resonance frequency and the change in applied mass^45^.


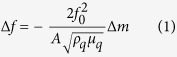


where *∆f* and *f*_*o*_ are the frequency shift and the resonant frequency of FBAR, *μ*_*q*_ and *ρ*_*q*_ are the shear modulus and the density of piezoelectric material, *A* is the area of the plate and *∆m* is the deposited mass. In the Sauerbrey equation, the sensitivity of an FBAR sensor (*∆f*/*∆m*) is in proportion to square of *f*_*o*_, which means the sensitivity of 4.44 GHz FBAR used in this work is orders of magnitude higher than SAW devices and QCM.

### Surface modifications and characterizations

In this work we applied nine different SAMs as gas sensitive materials to coat the FBAR sensor: (3-aminopropyl) dimethylethoxysilane (APDMES), (3-aminopropyl) diethoxymethylsilane (APDES), (3-aminopropyl) triethoxysilane (APTES), (3-glycidyloxypropyl) trimethoxysilane (GPTES), trimethoxy (octadecyl) silane (OTES), (3-bromopropyl) trichlorosilane (BPTS), trichloro (1H,1H,2H,2H-perfluorooctyl) silane (PFDTS), methyl-PEG4-NHS ester and methyl-PEG12-NHS ester. Due to the sub-nanoscale size of silane molecules, it is hard to quantificationally measure the coverage of the silane monolayer. To our knowledge, the YES-LabKote heated chamber is the most reliable and repeatable method for vapor deposition. And the layer modified by vapor deposition method is known to be superior uniform and consistent than liquid phase deposition^46^.

**Figure 2:**
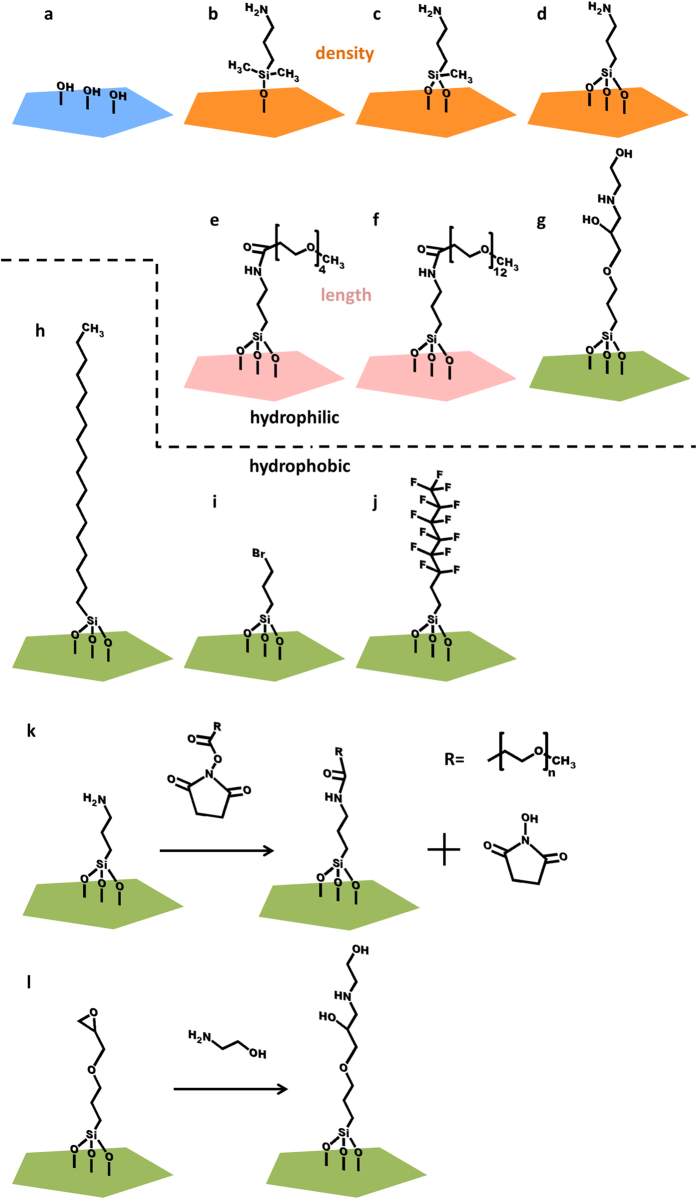
Schematic illustration of the FBAR surfaces modified with SAMs: (**a**) hydrophilic surface after oxidized in exposure to air plasma, (**b**) APDMES, (**c**) APDES, (**d**) APTES, (**e**) methyl-PEG4-NHS, (**f**) methyl-PEG12-NHS, (**g**) GPTES, (**h**) OTES, (**i**) BPTS and (**j**) PFDTS, and the modification process of (**k**) methyl-PEGn-NHS and (**l**) GPTES.

We separated the SAMs into three characteristic groups in terms of their hydrophilic-hydrophobic property, functional group density and chain length. For hydrophilic-hydrophobic property, APDMES, APDES, APTES, methyl-PEG4-NHS, methyl-PEG12-NHS and GPTES are assumed to be hydrophilic while OTES, BPTS and PFDTS are assumed to be hydrophobic. For studies of density of functional groups, three different amino silanes of APDMES (mono-ethyoxyl), APDES (diethoxyl) and APTES (triethoxyl) are chosen due to their different binding capacity with hydroxyl groups on the sensor surface which will result different amine densities. And for chain length effects, methyl-PEG4-NHS and methyl-PEG12-NHS are chosen which have different PEG chain length. Figure 2 shows the chemical structures of the SAMs. Fourier transform infrared spectroscopy (FT-IR) under vacuum was used to confirm the chemical functionalization of the monolayers. Figure S3 shows the FT-IR spectra of the modified monolayers on the AlN substrates.

Three kinds of amino-silane layers were prepared on glass slides through gas phase silane chemistry under the same conditions. The employed amino-silane reagents include APDMES, APDES and APTES respectively. Due to their chemical structure variations (APDMES (mono-ethyoxyl), APDES (diethoxyl) and APTES (triethoxyl)), they showed different binding capacities to the surface hydrocyl group, thus will induce different surface amine densities. Here, we applied 5(6)-Carboxytetramethylrhodamine N-succinimidyl ester (TAMRA) dye as fluorophores to covalent bind with amine and indirectly quantify the surface amine densities. After silanization, each glass slide was immersed into the solution of TAMRA dye in ethanolamine and dimethyl sulfoxide (DMSO) (1 mg ml^−1^) for two hours. Fluorophores TAMRA was spontaneously reacted with amines. Accordingly, amine density can be characterized by the fluorescence intensity.

Though the fluorescent intensity can be measured by the fluorescent images, the result is strongly determined by the focus effect and selected regions. More accurate way to determine the fluorescent intensity is from the fluorescent spectroscopy. Here, in this work we used a microplate reader to measure the fluorescent intensity using the transmission model thus the overall fluorescent intensity of the glass slide is averaged. Actually the obtained intensity results are agreed well with the fluorescence microscopy images (S9 in Supporting Information). Table 1 presents the fluorescence intensity results by the Multimode Microplate reader. Compared with blank control group, TAMRA modified glass slides show significant increase in fluorescence intensity, indicates a successful fluorophore binding. And for the comparison of three SAMs, the fluorescence intensity is, in descending order, APDMES, APDES and APTES, indicating the order of amine density. The results further confirm the successful amine surface modifications through the silane chemistry and the results are corresponding to other studies as well^47^.

In order to evaluate the hydrophilic-hydrophobic properties of the functionalized monolayers through silane chemistry, water contact angles (CAs) on each AlN substrate after chemical treatment were measured. Figure S2 presents the CA results of nine SAMs. Obviously, the results show the hydrophilicity of methyl-PEG4-NHS, methyl-PEG12-NHS and GPTES monolayers and the hydrophobicity of OTES, BPTS and PFDTS monolayers. Besides, the amino group monolayers (APDMES, APDES and APTES) show weak hydrophilicity.

For the three amino SAMs, hydrophilicity is assumed to have positive correlation with the density of hydrophilic amino group, yet the result is not so. This is due to the hydrophobic methyl group on the side chain of APDMES and APDES. For APDES, as the introduction of methyl group, the total hydrophilicity of the amino-terminal monolayer is reduced. For APDMES, as the amine density becomes larger, the hydrophilicity is increased. The difference of hydrophilic-hydrophobic properties between these surfaces indicates the diversity of FBAR array, which contributes to the distinguishability of the e-nose system.

### Real-time gas sensing results of FBAR arrays

**Figure 3:**
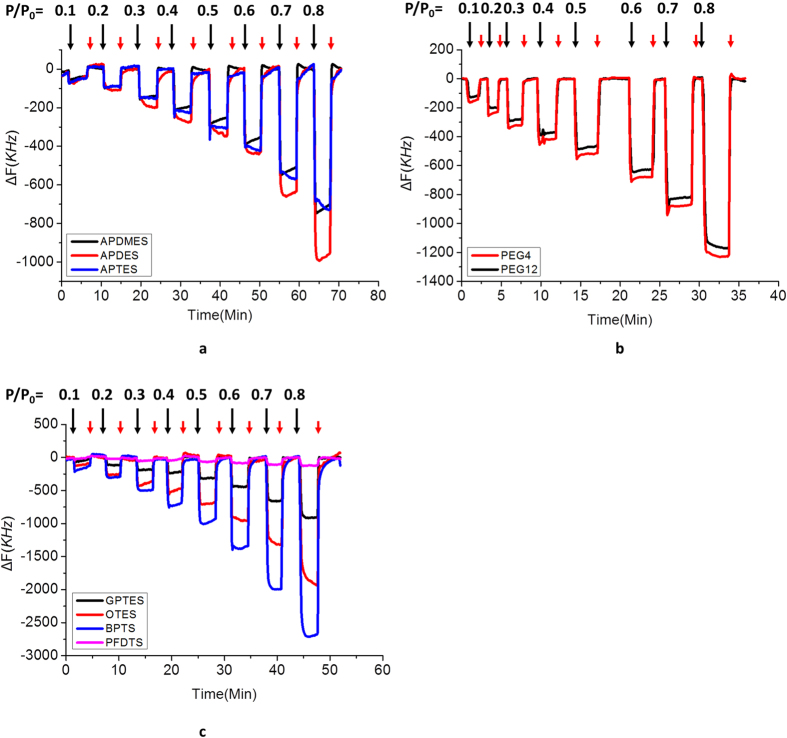
The adsorption-desorption process of three FBAR arrays: (**a**) APDMES, APDES and APTES, (**b**) methyl-PEG4-NHS and methyl-PEG12-NHS, (**c**) GPTES, OTES, BPTS and PFDTS at eight different gas partial pressure for eight consecutive cycles, nitrogen flow (red arrows) and injection of VOCs (IPA, black arrows).

After silanization, the functionalized FBARs were wire-bonded to evaluation boards, which were packaged in glass chamber with a gas intet and outlet. Nine functionalized FBARs were divided into three groups for detection (group A: APDMES, APDES and APTES, group B: methyl-PEG4-NHS and methyl-PEG12-NHS, and group C: GPTES, OTES, BPTS and PFDTS). Figure 3 shows the real-time frequency responses of three groups of FBAR arrays exposed to IPA vapor with increased gas partial pressures. Before VOC injection, FBARs in the glass chamber were firstly flushed with pure nitrogen to reach a stable baseline. Once exposed to VOCs, resonant frequency decreased which is due to the molecule adsorption. When the gas flow switched from VOCs to nitrogen, the frequency increased due to molecule desorption. It is clearly showed from Fig. 3 that the adsorption-desorption of VOCs on different SAMs is a completely reversible process which is benefiting from the monolayer surface modifications. This is a very important issue for e-nose applications since the incomplete gas molecule desorption will cause the malfunctions of the e-nose system (*e,g,* Electronic Nose Poisoning^48^). The results also show that adding the gas partial pressure will increase the amount of the VOCs adsorptions. The real time gas detections of other gas molecules (acetone, NPA, ethanol, and Methanol) on SAMs are shown in the Supplementary.

At a constant gas pressure, the frequency shifts of different monolayers to a certain VOC are discriminative, which indicates that these values can be utilized to create a concentration-dependent histogram as VOC’s unique code (*i.e.*, a typical fingerprint). Compared with hydrophilic SAMs, two hydrophobic SAMs, OTES and BPTS, get higher responses. Similarly, between three amine monolayers, the more hydrophobic APDES shows higher response to VOC molecules. It is likely due to the hydrophobic–oleophilic character of these SAMs. An exception is the PFDTS monolayer. Because of its special repellency property^49^, fluorinated coating shows the lowest response to all the VOCs.

### Adsorption isotherms of VOCs on SAMs

In principle, the direct sensor responses (frequency shift) can be used as fingerprints to distinguish different VOCs since each VOC has different fingerprint patterns however, such fingerprint pattern is not the same at different gas concentrations, thus the concentration dependent fingerprint pattern is hard to be used to discriminate an unknown gas target. Here, we demonstrate the concept to create a concentration-independent fingerprint library by using the fitting results from the gas adsorption isotherms and desorption kinetics.

**Figure 4:**
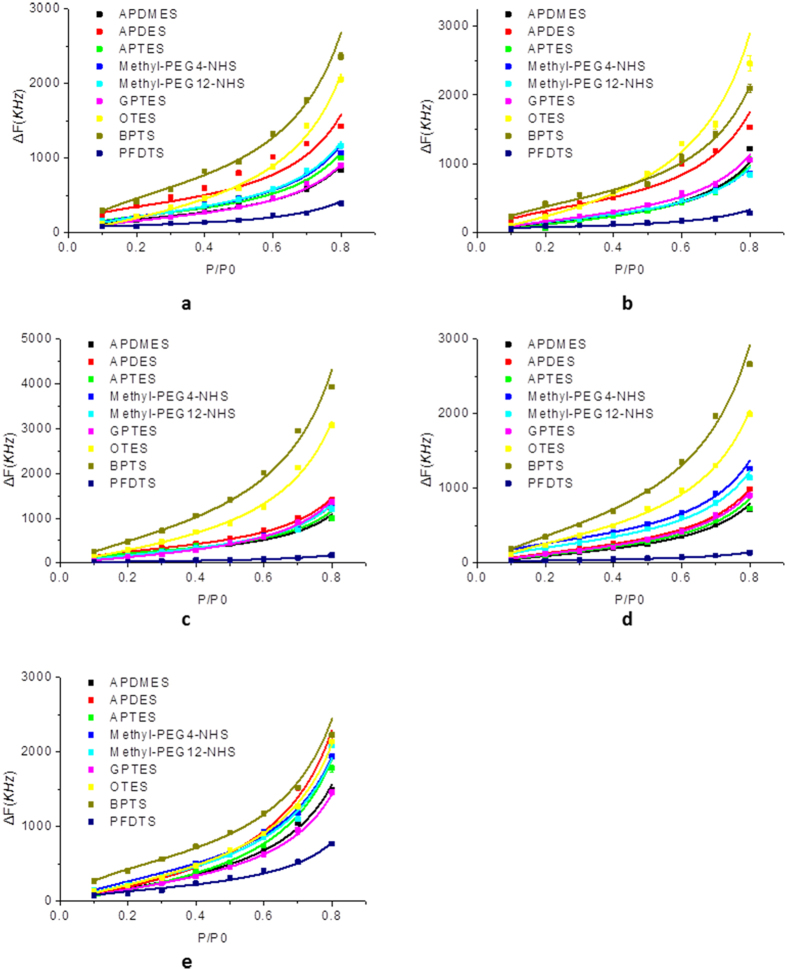
Adsorption isotherms of VOCs: (**a**) methanol, (**b**) ethanol, (**c**) NPA, (**d**) IPA, and (**e**) acetone on different monolayers. The solid lines are the fitting results using the BET equation.

Based on the frequency shifts at different gas partial pressures, adsorption isotherms of the each VOC on nine different SAMs can be obtained accordingly. As shown in Fig. 4, the adsorption isotherms all fit well with Brunauer-Emmett-Teller (BET) equation^50^,^51^, which is the typical model of multilayer gas physisorptions.


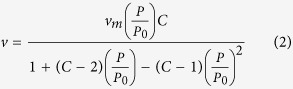


where *v*, *v*_*m*_, *p* and *p*_*o*_ are adsorption capacity, monolayer adsorption capacity, vapor pressures and saturated vapor pressure of VOC respectively, and *C* is the adsorption energy constant. It can be indicated that the gas-solid interaction of VOCs on SAMs is not monolayer adsorption, gas molecule accumulation still occurs on the saturated surface. Relied on the fitting results, two concentration-independent parameters, *v*_*m*_ and *C* can be obtained for the fingerprint library.

### Sorption kinetics of VOCs on SAMs

**Figure 5:**
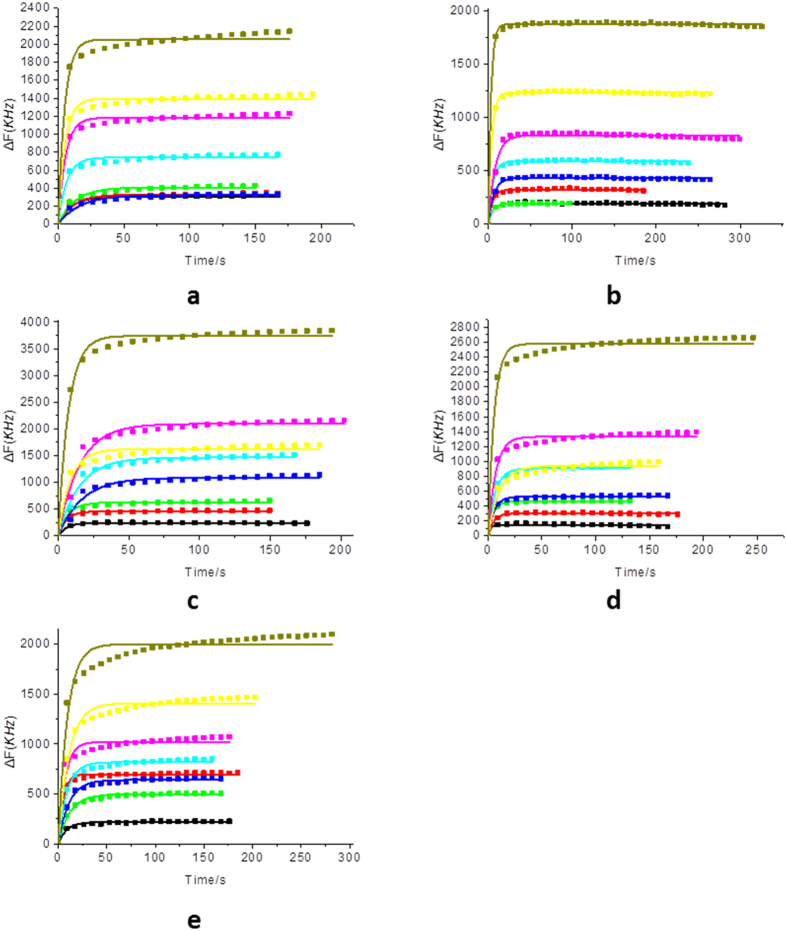
Desorption process of VOCs: (**a**) methanol, (**b**) ethanol, (**c**) NPA, (**d**) IPA, and (**e**) acetone on BPTS monolayer at different gas partial pressure. The solid lines are the fitting results using the JMA equation.

In order to fully understand the sorption process of gas-surface interactions, the following Johnson-Mehl-Avrami (JMA) equation^52^ is adopted to fit the desorption process of different VOCs on SAMs.


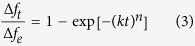


where *k* is the reaction rate, *n* is the reaction exponent and *t* is the reaction time. *∆f*_*t*_ and *∆f*_*e*_ represent the experimental frequency shift due to VOCs desorption at *t* and after reaching equilibrium. As shown in Fig. 5, the desorption process agrees well with the JMA equation. The obtained desorption rate constant, *k*, is concentration-independent. We here use the average value *k* of eight gas partial pressures as another parameter in the fingerprint library.

### Concentration-independent fingerprint library of VOCs

**Figure 6:**
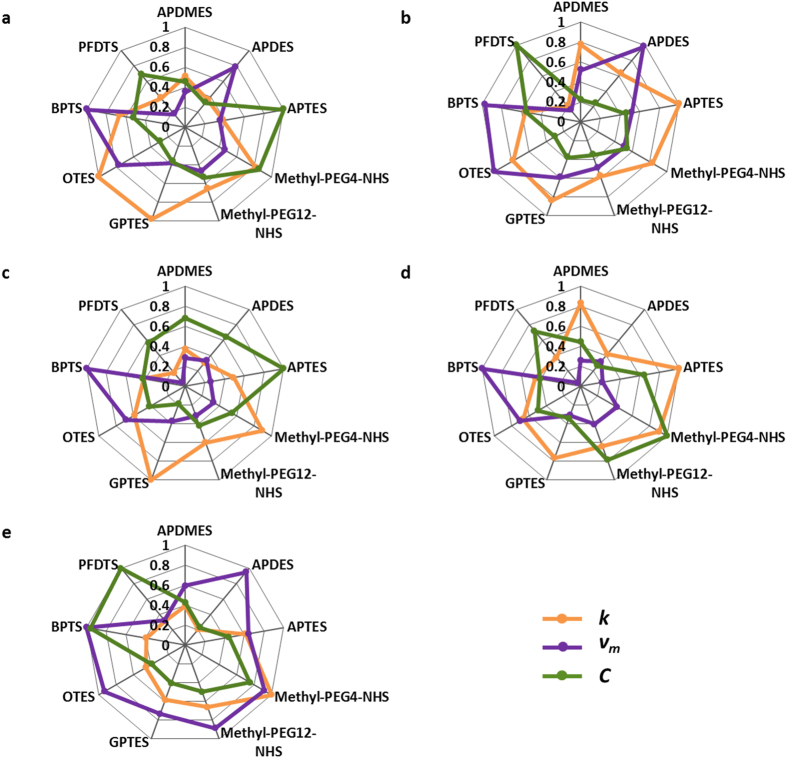
Fingerprint library of five VOCs: (**a**) methanol, (**b**) ethanol, (**c**) NPA, (**d**) IPA, and (**e**) acetone.

Based on the three above-mentioned parameters of nine SAMs for each VOC, a concentration-independent fingerprint library is built. Figure 6 represents the three normalization parameters used to contribute to the fingerprint library to distinguish each kind of VOCs. The concentration-independent fingerprint library provides a solid method for the discrimination for the analyte with unknown concentrations. By simple dilution of an unknown target, the absorption isotherms and desorption kinetic parameters are obtained and the fingerprint pattern can be constructed accordingly, thus it can be compared to the known fingerprint library to tell the gas content while the gas concentration can be directly determined from the sensor response. Besides, the nature of each SAM is displayed in the fingerprint library, which can serve as an information database for gas-sensitive materials selection.

## Discussion

The amount of the VOC adsorptions (frequency shifts of FBAR sensors) is determined by the oleophilic (or oleophobic) character of the SAMs. The monolayer adsorption capacity (*v*_*m*_) follows this rule as well. The value *v*_*m*_ of oleophobic PFDTS is the lowest while the values of two oleophilic SAMs are the highest. It can be concluded that the affinity of gas-surface interactions is wettability-dependent. APDMES, APDES and APTES are chosen to compare their amine densities effects on the VOCs adsorptions. However, due to the impact of hydrophilic-hydrophobic property, the more hydrophobic APDES has the highest *v*_*m*_ value while its amine density ranks in the middle. Hence, with similar hydrophilicity, the contrast of APDMES (highest amine density) and APTES (lowest amine density) can be utilized to analyze the effect of amine density as the impact of wettability is enormously reduced. From these results, it can be summarized that *v*_*m*_ of APDMES is roughly close to APTES. Methyl-PEG4-NHS ester and methyl-PEG12-NHS ester are chosen as their different PEG chain length. From the results, molecule chain length has tiny effect on VOCs adsorption capacity. Thus, it seems that general ‘wettability’ is the major factor for VOCs adsorption capacity.

Desorption rate (*k*) is another wettability-dependent parameter. Results show that in the majority, the value *k* of hydrophilic SAMs is higher than that of hydrophobic SAMs. It is likely that for hydrophilic surface, the desorption process is faster to reach equilibrium due to its oleophobicity, while hydrophobic-oleophilic surface is not. On the other hand, higher group density and longer chain length both reduce the desorption rate, indicates that the “high number of molecular surface” (surface modified by SAMs with high group density as well as long chain length) reduces the escape ability of VOC molecules.

**Figure 7:**
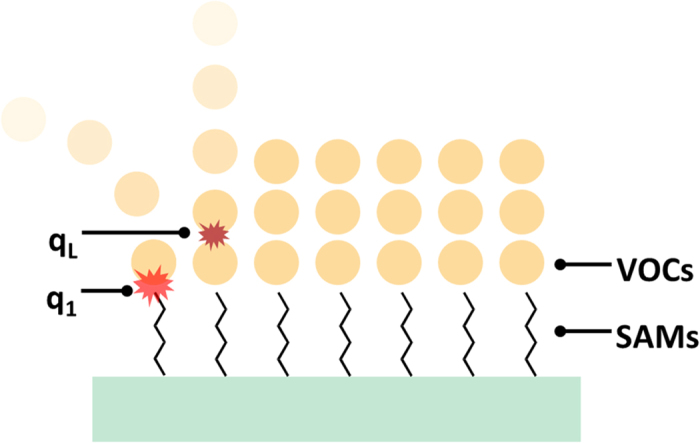
The difference between adsorption energy of first layer and subsequent layers.

In the BET model, the constant *C* is related to the difference between adsorption energy of first layer and all subsequent layers which is given by


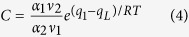


where *α*_*1*_ and *α*_*2*_ are the condensation coefficients for the first and subsequent layers, *ν*_*1*_ and *ν*_*2*_ are the frequencies of oscillation of molecules in the first and subsequent layers, *q*_*1*_ is the heat of adsorption in the first layer on the surface and *q*_*L*_ is the heat of condensation of subsequent layers (Fig. 7). The presence of methyl group is found to have a close relationship with the value of *C*. For example, in the three amino SAMs, the *C* value of APTES (without any methyl groups) is significantly higher than that of APDMES and APDES (with methyl group on the side chain). It indicates that for approximate condensation heat of subsequent VOCs, methyl group lowers the adsorption energy between amino SAMs and VOCs. The probable explanation could be due to the electronegativity difference between nitrogen and carbon atom, VOCs can be adsorbed by hydrogen bond between amine and hydroxyl of alcohols or carbonyl of acetone, while for methyl site, the force is more likely to be Van der Waals force which is relatively smaller. In a similar way, the *C* value of OTES with alkyl carbon structure is the lowest in the majority.

With higher operating frequency and sensitivity, FBAR shows potential to measure trace levels of VOCs. In order to meet this demand, advanced vapor recognition materials (*e.g.* polymer coatings) can be used as sensitive layers on FBAR devices. The selection of vapor recognition materials will benefit from the fingerprint library of various surface chemical groups. Besides, a pre-concentrator is normally integrated before the gas sensor array to separate and concentrate various VOCs thus improves the limit of detection of the e-nose system.

In this work, 4.44-GHz FBARs were successfully fabricated by a standard microelectromechanical system (MEMS) fabrication process. Multiple chemical functionalization was achieved by vapor phase deposition of silylating reagent under reduced pressure in a heated chamber. Different functionalized FBARs were packaged and integrated to constitute e-nose sensor arrays. Different concentrations of VOCs (methanol, ethanol, NPA, IPA and acetone used in this work) were applied by a home-made gas delivery system. Adsorption isotherms of each vapor on nine functional groups were successfully obtained. Accordingly, adsorption energy constant and monolayer adsorption capacity were determined by fitting the adsorption isotherms to a BET equation. Desorption rate was determined by fitting a JMA equation. Relied on the three parameters, concentration-independent fingerprints were well established. Such concentration- independent fingerprint library facilitates the discriminations of target VOCs using the sensor array, leading to a more reliable method for VOCs detections. Besides, effects of characteristics of the surface chemical groups are analyzed to study the gas-surface interaction which demonstrates such sensor array can be used for gas-sensitive materials selections.

## Methods

### Chemicals

APDMES, APDES, APTES, GPTES, OTES, BPTS, PFDTS, methyl-PEG4-NHS ester, methyl-PEG12-NHS ester and TAMRA were purchased from Aladdin Industrial Corporation without further purification. VOCs (methanol, ethanol, NPA, IPA and acetone), DMSO utilized in this work were purchased from Tianjin Real&Lead Chemical Corporation and the purity all reached HPLC.

### Device fabrication

The 4.44 GHz FBARs in this work (shown in Fig. S8) were conveniently fabricated by a standard MEMS fabrication process as described before^53^. In brief, an air cavity was initially generated on a single-side polished silicon wafer by deep reactive ion etching (DRIE). The air cavity was subsequently filled with phosphosilicate glass (PSG) used as sacrificial layer by chemical vapor deposition (CVD). A sandwiched structure, Mo, AlN and Mo were sequentially deposited on the substrate as bottom electrode, piezoelectric layer and top electrode, respectively. A passivation layer (AlN) was then deposited for the protection from oxidation. Finally, the wafer was immersed in diluted HF solution to remove PSG in the cavity.

### Device functionalization

FBARs were firstly oxidized in exposure to air plasma for 5 min to form hydrophilic interface by a plasma cleaner (YZD08-2C, SAOT, China). Silanization was achieved by vapor phase deposition of silylating reagent under reduced pressure in a heated chamber (YES-LabKote, Yield Engineering Systems, USA). PEG coating were achieved by exposing APTES functionalized devices into methyl-PEG4-NHS and methyl-PEG12-NHS solution (1 mg ml^−1^, Na_2_CO_3_, pH 8) for two hours, respectively. GPTES functionalized device was further reacted with aqueous ethanolamine solutions (20%) for two hours to form hydroxyl terminated SAMs. Functionalized FBARs were worked and stored in a nitrogen environment (*e.g.* glove box) at room temperature for the protection of SAMs from oxidation damage and hydrolysis damage. Such devices can be kept for half year without notable degradation.

### Surface characterization

The fluorescent intensity was characterized by a Multimode Microplate reader (Varioskan LUX, Thermo Fisher Scientific, Vantaa, Finland). Silane monolayers coated on AlN substrates were characterized by contact angle (CA) measurements (JC2000DM, Zhongchen, China) and Fourier transform infrared (FT-IR) spectrometer (Vertex 70v, Bruker Optics, Germany).

### VOCs delivery system

A dual-line VOCs delivery system was utilized in this work. Vapor of the VOC line was delivered by bubbling 99.999% pure carrier N_2_ gas into VOC liquid. The vapor bubbler was kept in good contact with a temperature controlled oil bath, maintained at 26 °C, in this case, no obvious drifts were found in the continuous process of experiments. Different ratios of vapor partial pressure to saturated vapor pressure (P/P_0_) were achieved by adjusting the flow velocity of N_2_ from the dilution line^54^. And the flow velocity was monitored by a mass flow controller (MFC, 5850e, Brooks, USA). The total flow velocity varied from 250 sccm to 2000 sccm. Based on our verified experiment, it was found that the flow rate had a negligible impact on sensor’s performance (Supplementary Fig. S9).

### VOCs testing system

Functionalized FBARs were wire-bonded to evaluation boards, which were packaged in glass chambers (10 mL) and connected to a network analyzer (E5071C, Agilent, USA). Real-time sensing data were recorded by a MATLAB program.

## Additional Information

**How to cite this article**: Chang, Y. *et al.* Detection of Volatile Organic Compounds by Self-assembled Monolayer Coated Sensor Array with Concentration-independent Fingerprints. *Sci. Rep.* **6**, 23970; doi: 10.1038/srep23970 (2016).

## Supplementary Material

Supplementary Information

## Figures and Tables

**Figure 1:**
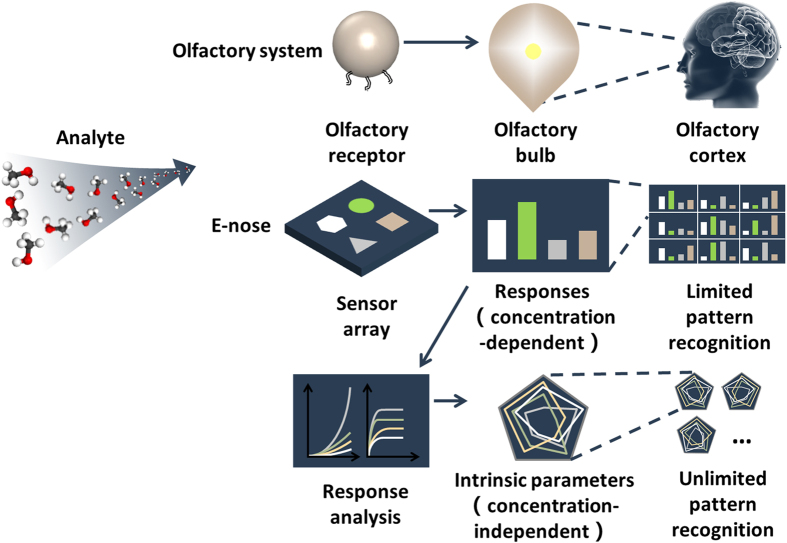
Schematic illustration of the mechanism of olfactory system and e-nose system (both concentration-dependent and concentration-independent).

**Table 1:** Fluorescence intensity results of the amino group SAMs.

Relative fluorescence units	APDMES	APDES	APTES
Glass slide 1	7.456	6.841	3.714
Glass slide 2	7.988	4.427	3.953
Glass slide 3	5.035	4.551	3.107
Blank control	0.3222	0.2085	0.3926
