# Supplementary material for: Detection of Volatile Organic Compounds by Self-assembled Monolayer Coated Sensor Array with Concentration-independent Fingerprints
Source: Sci Rep. 2016 Apr 5;6:23970. doi: 10.1038/srep23970 (PMC4820718; doi:10.1038/srep23970)
Supplement: Supplementary Information [file srep23970-s1.pdf]

Supporting Information

Detection of Volatile Organic Compounds by Self-assembled Monolayer Coated Sensor Array with Concentration-independent Fingerprints

Ye Chang,[#] Ning Tang,[#] Hemi Qu, Jing Liu, Daihua Zhang, Hao Zhang, Wei Pang, Xuexin Duan[*]

State Key Laboratory of Precision Measuring Technology & Instruments, College of Precision Instrument and Opto-electronics Engineering, Tianjin University, Tianjin 300072, China

# These authors contributed equally to this work.

* Corresponding Authors.

Tel.: +86-22-27401002

E-mail: xduan@tju.edu.cn

1. Amine-NHS reaction

Figure S1 illustrates the reaction process of 5(6)-Carboxytetramethylrhodamine N-succinimidyl ester (TAMRA) with (3-aminopropyl) triethoxysilane (APTES) modified FBAR for amine density characterization

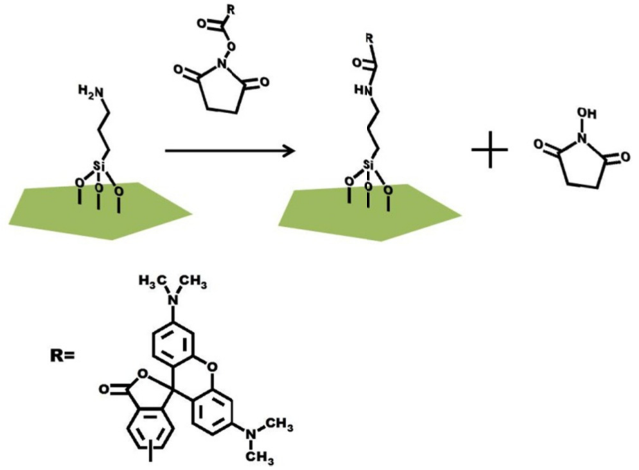

Figure S1. Reaction process of amine with TAMRA.

2. Contact angles measurement

Figure S2 shows the CA results of nine SAMs.

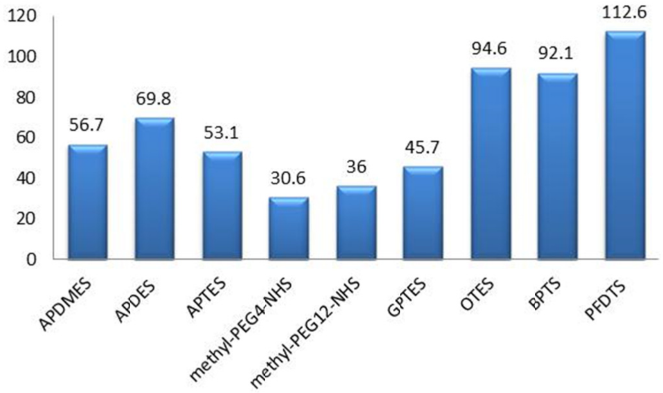

Figure S2. CA results of AlN substrates after silane chemistry of nine SAMs.

3. Fourier transform infrared spectroscopy measurement

Figure S3 shows the FT-IR spectra of the modified monolayers on the AlN substrates. APTES-modified and OTES-modified monolayer spectrums show reflection peaks at $1546cm^{-1}$ and $2860{\sim}2930cm^{-1}$ due to the vibration of NH_2 and the stretch of CH_2, respectively. Meanwhile, Methyl-PEG-NHS-modified and GPTES-modified monolayer spectrums peak at $1103cm^{-1}$ and $1265cm^{-1}$, which is attributed to stretching of the functional group of C-O . The characteristic vibration of the bromine end-group at $692cm^{-1}$ confirms the BPTS monolayer. Additionally, the C-F vibration at $1260cm^{-1}$ was assigned to the PFDTS functionalization monolayer, indicating the formation of fluoride group of the PFDTS after silanization.

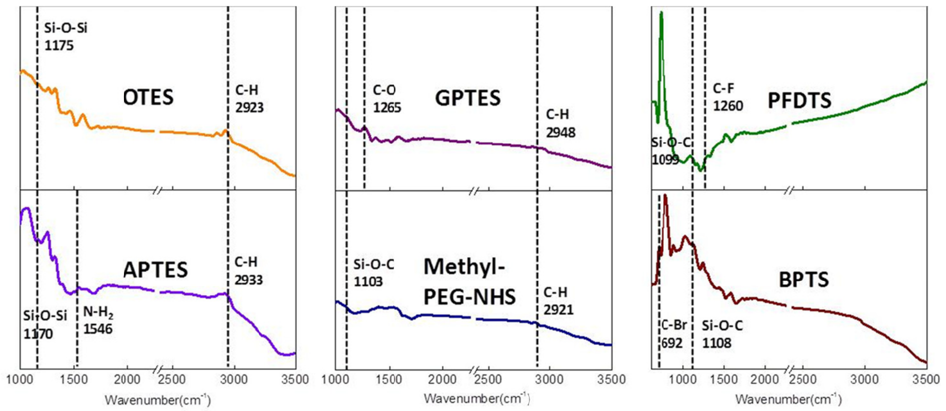

Figure S3. Surface IR spectra of the SAMs on AlN substrates.

4. VOCs detection setup

Figure S4 shows the VOCs detection setup used in this work. The setup consists of two parts, the VOCs delivery system and the VOCs testing system. In the VOCs delivery system, vapor of the VOC line was delivered by bubbling 99.999% pure carrier N2 gas into VOC liquid. Different gas partial pressures were achieved by adjusting the flow velocity of N2 from the dilution line. The flow velocity was monitored by the mass flow controller. For exhausts treatment, a VOCs absorber filled with ethanol was added at the end of the system. In the VOCs testing system, functionalized FBARs were wire-bonded to evaluation boards, which were packaged in glass chambers and connected to a network analyzer.

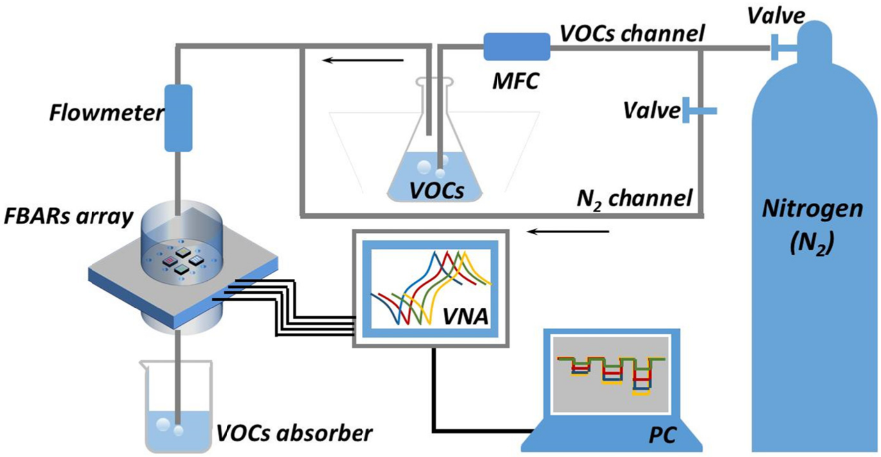

Figure S4. The VOCs detection setup.

5. Real-time frequency responses of FBAR arrays

Figure S5-7 shows the real-time frequency responses of FBAR arrays in exposure to methanol, ethanol, NPA and acetone vapors respectively at eight different concentrations.

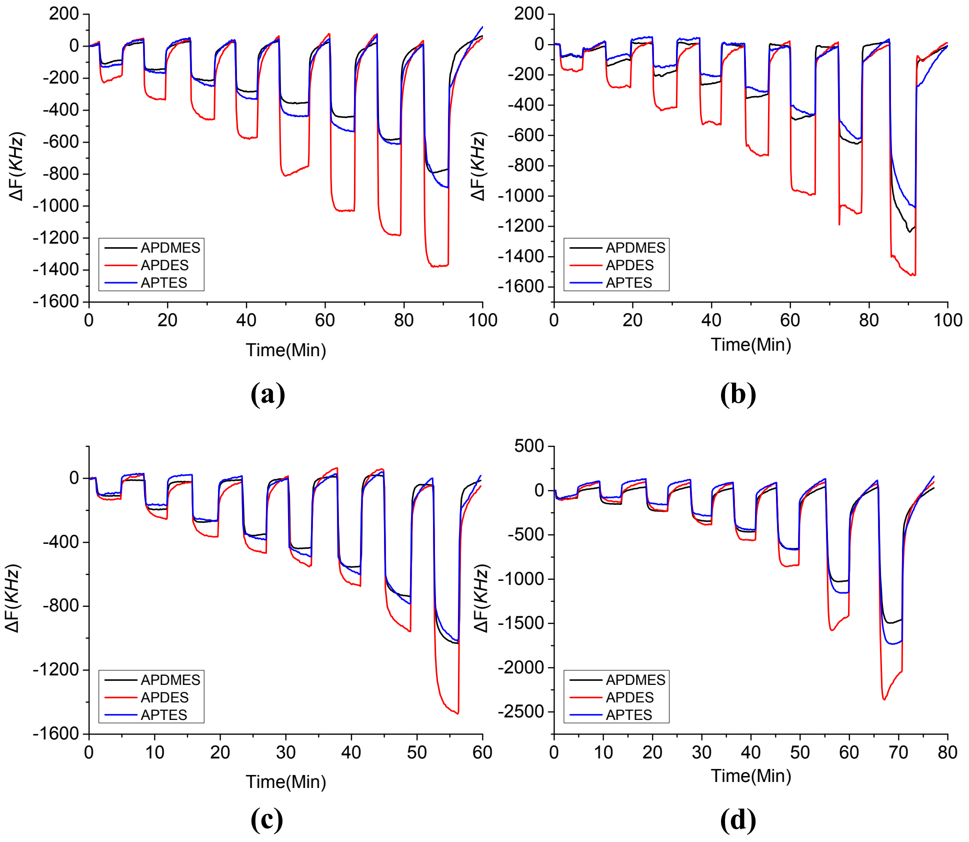

Figure S5. Real-time frequency responses of the FBAR arrays functionalized with APDMES, APDES and APTES in exposure to (a) methanol, (b) ethanol, (c) NPA and (d) acetone vapors respectively at eight different concentrations.

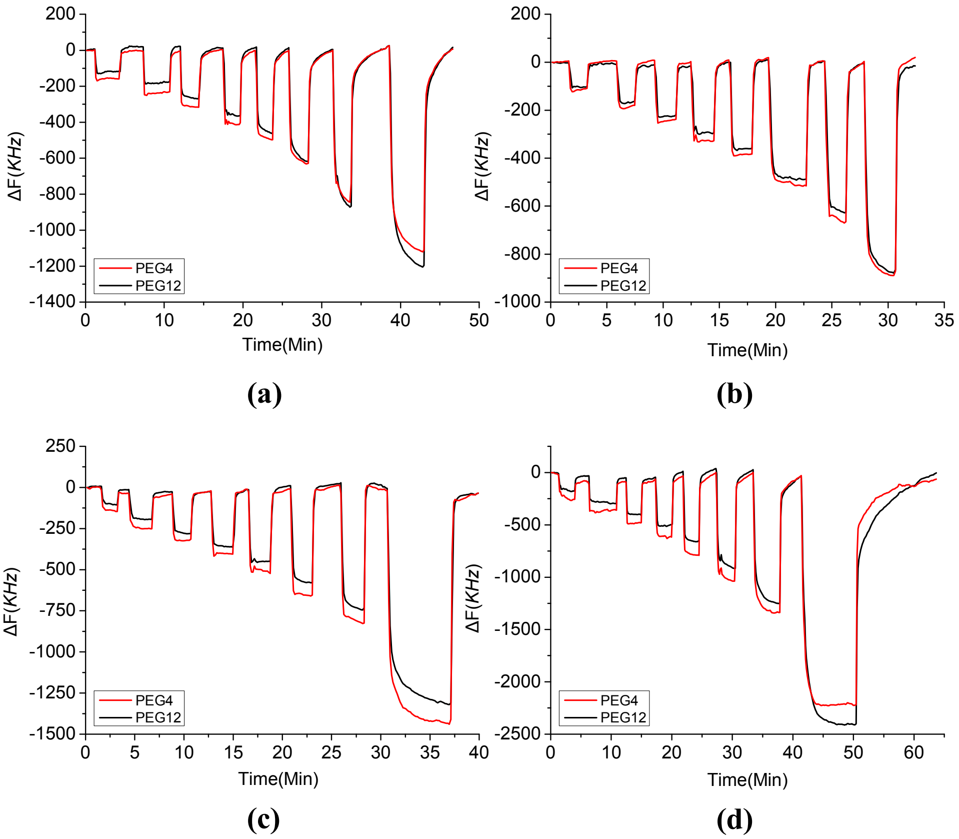

Figure S6. Real-time frequency responses of the FBAR arrays functionalized with methyl-PEG4-NHS and methyl-PEG12-NHS in exposure to (a) methanol, (b) ethanol, (c) NPA and (d) acetone vapors respectively at eight different concentrations.

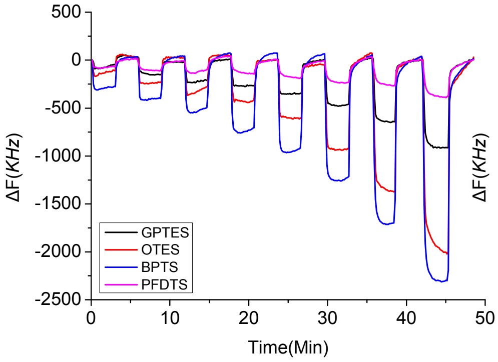

(a)

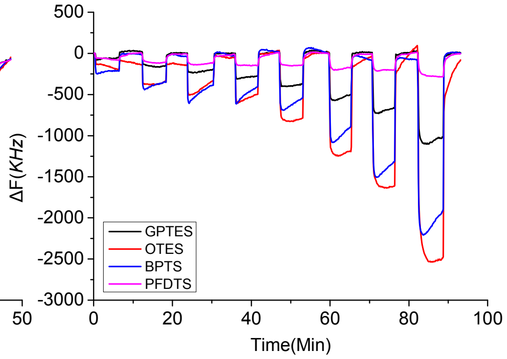

(b)

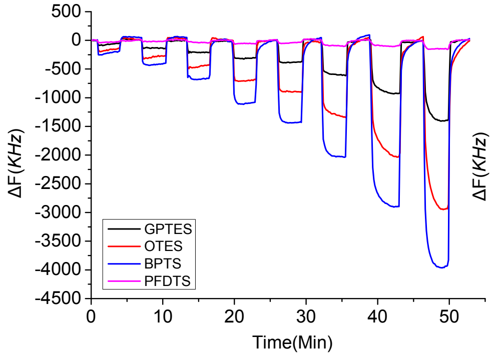

(c)

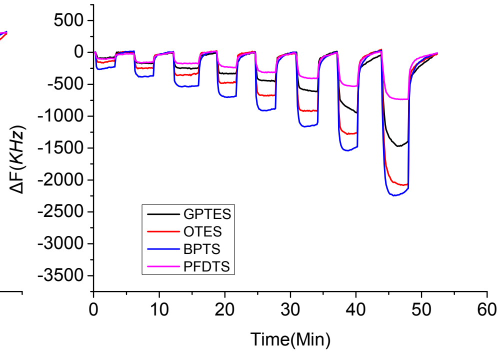

(d)

Figure S7. Real-time frequency responses of the FBAR arrays functionalized with GPTES, OTES, BPTS and PFDTS in exposure to (a) methanol, (b) ethanol, (c) NPA and (d) acetone vapors respectively at eight different concentrations.

6. Fitting results of adsorption isotherms

Adsorption isotherms of five VOCs to nine SAMs were fitted with BET equation. Table S1 lists the fitting results.

Table S1. Fitting results of adsorption isotherms.

VOC	Methanol			Ethanol			NPA		
SAMs	v_m	C	r^2	v_m	C	r^2	v_m	C	r^2
APDMES	175.8668	6.75968	0.9946	67.24171	11.33556	0.96086	237.249	6.45488	0.98538
APDES	393.7338	4.70486	0.98089	161.8313	5.23239	0.99918	279.3458	6.08471	0.99179
APTES	177.4176	14.99026	0.99289	232.3056	2.47531	0.9972	217.1876	9.48507	0.96804
PEG4	231.8277	12.77046	0.99482	220.3477	4.01286	0.97772	276.6123	5.1279	0.99933
PEG12	237.2227	8.05648	0.99609	225.542	6.09021	0.98967	265.9447	3.99252	0.99889
GPTES	191.5279	5.51511	0.99323	215.9976	4.33559	0.98609	321.4467	1.81317	0.99905
OTES	383.856	4.34145	0.97656	394.2374	6.25385	0.99359	568.6147	3.8841	0.99388
BPTS	502.9128	7.82923	0.99519	439.1493	2.66965	0.99	836.3522	4.07558	0.98776
PFDTS	85.00887	10.27118	0.9928	449.4547	3.36969	0.98839	36.87076	5.34105	0.95366

VOC	IPA			Acetone		
SAMs	v_m	C	r^2	v_m	C	r^2
APDMES	159.5244	4.54017	0.98965	251.8043	3.54231	0.99942
APDES	200.34674	2.70269	0.9993	404.27549	1.91198	0.99039
APTES	138.72842	6.64317	0.99598	274.28098	3.70152	0.99044
PEG4	263.67588	10.29638	0.99237	388.98473	6.21751	0.99488
PEG12	254.66766	8.10887	0.98996	376.58567	4.13778	0.99202
GPTES	195.47399	3.53581	0.9923	245.70181	3.3742	0.99452
OTES	387.81368	5.05927	0.99144	395.61681	3.19108	0.99799
BPTS	556.32641	4.25224	0.98105	425.35995	7.98229	0.99554
PFDTS	27.15954	7.38894	0.99577	136.90933	8.31037	0.99385

7. Fitting results of desorption process

Desorption processes of five VOCs to nine SAMs were fitted with JMA equation. Table S2 lists the fitting results.

Table S2. Fitting results of desorption process.

VOC	Methanol		Ethanol		NPA		IPA		Acetone	
	k	S	k	S	k	S	k	S	k	S
APDMES	0.1189	0.03092	0.22119	0.08681	0.12964	0.04215	0.30263	0.1153	0.11806	0.02082
APDES	0.0834	0.03254	0.17991	0.09442	0.10621	0.00992	0.15183	0.03993	0.06144	0.01469
APTES	0.09077	0.02599	0.28644	0.08406	0.17291	0.02727	0.36478	0.12635	0.18646	0.04215
PEG4	0.19272	0.05493	0.2381	0.03142	0.31233	0.13881	0.33169	0.12909	0.30285	0.10798
PEG12	0.1555	0.03792	0.16642	0.03979	0.21166	0.07365	0.23433	0.03308	0.20022	0.06201
GPTES	0.2317	0.03362	0.24077	0.10242	0.3506	0.17111	0.27949	0.11055	0.17721	0.09037
OTES	0.2357	0.04648	0.22387	0.07978	0.20449	0.06314	0.24248	0.06638	0.13728	0.045
BPTS	0.1581	0.05972	0.16033	0.06393	0.14777	0.01598	0.16749	0.03488	0.11949	0.02716
PFDTS	0.08886	0.01751	0.05797	0.01493	0.06085	0.02491	0.13655	0.09201	0.09492	0.03047

8. Normalization parameters of VOCs.

Table S3. Three normalization parameters of VOCs.

VOC		APDMES	APDES	APTES	PEG4	PEG12	GPTES	OTES	BPTS	PFDTS
	k	0.50445	0.35384	0.38511	0.81765	0.65974	0.98303	1	0.67077	0.377
Methanol	v_m	0.3497	0.78291	0.35278	0.46097	0.4717	0.38084	0.76327	1	0.16903
	C	0.45094	0.31386	1	0.85192	0.53745	0.36791	0.28962	0.52229	0.68519
	k	0.7722	0.62809	1	0.83124	0.58099	0.84056	0.78156	0.55973	0.20238
Ethanol	v_m	0.51686	0.97707	0.52147	0.50181	0.49026	0.59593	1	0.96985	0.14961
	C	0.21837	0.23551	0.46159	0.53727	0.35401	0.38248	0.29727	0.5517	1
	k	0.36977	0.30294	0.49318	0.89084	0.60371	1	0.58326	0.42148	0.17356
NPA	v_m	0.28367	0.334	0.25968	0.33074	0.31798	0.37573	0.67987	1	0.04409
	C	0.68053	0.6415	1	0.54063	0.42093	0.19116	0.4095	0.42968	0.5631
	k	0.82962	0.41622	1	0.90929	0.64239	0.76619	0.66473	0.45915	0.37434
IPA	v_m	0.25442	0.31952	0.22125	0.42052	0.40616	0.31175	0.69793	1	0.04332
	C	0.44095	0.26249	0.64519	1	0.78755	0.3434	0.49136	0.41298	0.71763
	k	0.38983	0.20287	0.61568	1	0.66112	0.58514	0.45329	0.39455	0.31342
Acetone	v_m	0.59198	0.95043	0.64482	0.91448	0.88533	0.73508	0.93008	1	0.32187
	C	0.42625	0.23007	0.44541	0.74816	0.49791	0.40602	0.38399	0.96052	1

9. Fluorescence microscopy images.

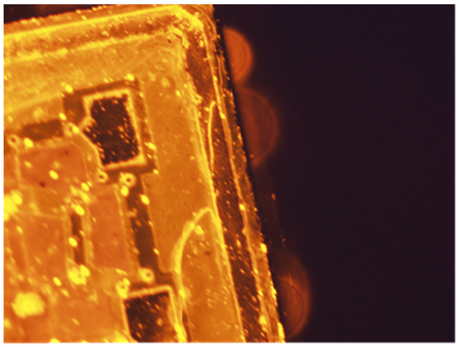

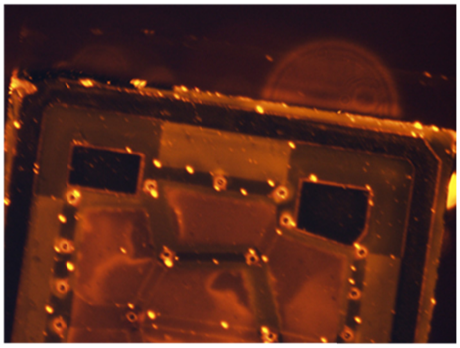

(a) APDMES

(b) APDES

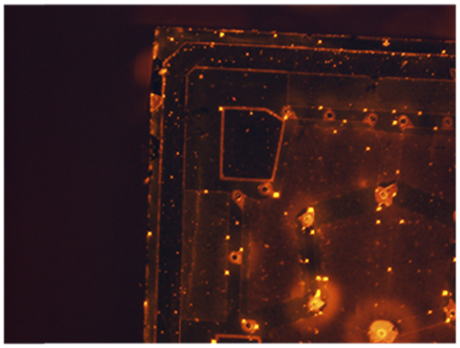

(b) APTES

Figure S8. The fluorescence microscopy images of APDMES, APDES and APTES reacted with fluorophores TAMRA. Relatively darker area corresponds to Au layer, while lighter area corresponds to silane-modified area.

10. The impact of flow rate on sensor's performance.

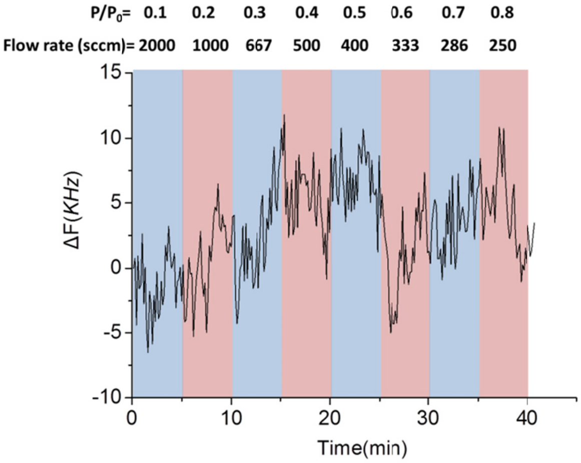

Figure S9. The real-time frequency of FBAR device at different flow rates of nitrogen. The flow rates correspond to the total flow rates of different concentrations of vapor in the detections (from 2000 sccm to 250 sccm).
